# Supplementary material for: Label-Free Optical Transmission Tomography for Direct Mycological Examination and Monitoring of Intracellular Dynamics
Source: J Fungi (Basel). 2024 Oct 26;10(11):741. doi: 10.3390/jof10110741 (PMC11595662; doi:10.3390/jof10110741)
Supplement: Supplementary file 1 [file jof-10-00741-s001.zip › jof-3251461-supplementary.pdf]

Journal of *Fungi*

MDPI

Article

Label-Free Optical Transmission Tomography for Direct Mycological Examination and Monitoring of Intracellular Dynamics

Eliott Teston [1], Marc Sautour [2,3], Léa Boulnois [4], Nicolas Augey [5], Abdellah Dighab [5], Christophe Guillet [5], Dea Garcia-Hermoso [6], Fanny Lanternier [6,7], Marie-Elisabeth Bougnoux [7], Frédéric Dalle [2,3], Louise Basmaciyan [2,3], Mathieu Blot [1,8], Pierre-Emmanuel Charles [1,9], Jean-Pierre Quenot [1,9], Bianca Podac [4], Catherine Neuwirth [10,11], Claude Boccara [12], Martine Boccara [13], Olivier Thouvenin [12] and Thomas Maldiney [1,14,*]

1 Lipness Team, Translational Research Center in Molecular Medicine– INSERM Joint Research Unit (CTM-UMR1231), University of Burgundy, 21000 Dijon , France
2 Department of Parasitology/Mycology, Dijon Bourgogne University Hospital, 21000 Dijon, France
3 Unité mixte de recherche Procédés Alimentaires et Microbiologiques (UMR PAM) A 02.102, Bourgogne Franche-Comté University, AgroSup Dijon, Dijon, France
4 Medical Biology Laboratory, William Morey General Hospital, 71100 Chalon-sur-Saône, France
5 LISPEN, Arts et Metiers Institute of Technology, 71100 Chalon-sur-Saône, France
6 Translational Mycology Research Group, Mycology Department, National Reference Center for Invasive Mycoses and Antifungals, Institut Pasteur, Paris Cité University, 75015 Paris, France
7 Necker-Enfants Malades Hospital, Assistance Publique-Hôpitaux de Paris, 75015 Paris, France
8 Infectious Diseases Department, Dijon Bourgogne University Hospital, 21000 Dijon, France
9 Department of Intensive Care Medicine, Dijon Bourgogne University Hospital, 21000 Dijon, France
10 Department of Bacteriology, University Hospital of Dijon, 21000 Dijon, France
11 UMR/CNRS 6248 Chrono-Environnement, Bougogne Franche-Comté University, 25000 Besançon, France
12 Institut Langevin, Ecole Supérieure de Physique et Chimie Industrielle de la ville de Paris, Université PSL, CNRS, 75005 Paris, France
13 Institut de Systématique, Evolution, Biodiversité– (ISYEB-UMR7205), Ecole Normale Supérieure, PSL Research University, 75005 Paris, France
14 Department of Intensive Care Medicine, William Morey General Hospital, 71100 Chalon-sur-Saône, France

Citation: Teston, E.; Sautour, M.; Boulnois, L.; Augey, N.; Dighab, A.; Guillet, C.; Garcia-Hermoso, D.; Lanternier, F.; Bougnoux, M.-E.; Dalle, F.; et al. Label-Free Optical Transmission Tomography for Direct Mycological Examination and Monitoring of Intracellular Dynamics. *J. Fungi* **2024**, *10*, 741. https://doi.org/10.3390/jof10110741

Academic Editor: Dong-Gun Lee

Received: 24 September 2024
Revised: 16 October 2024
Accepted: 23 October 2024
Published: 6 November 2024

Copyright: © 2024 by the authors. Licensee MDPI, Basel, Switzerland. This article is an open access article distributed under the terms and conditions of the Creative Commons Attribution (CC BY) license (https://creativecommons.org/licenses/by/4.0/).

Supplementary Materials:

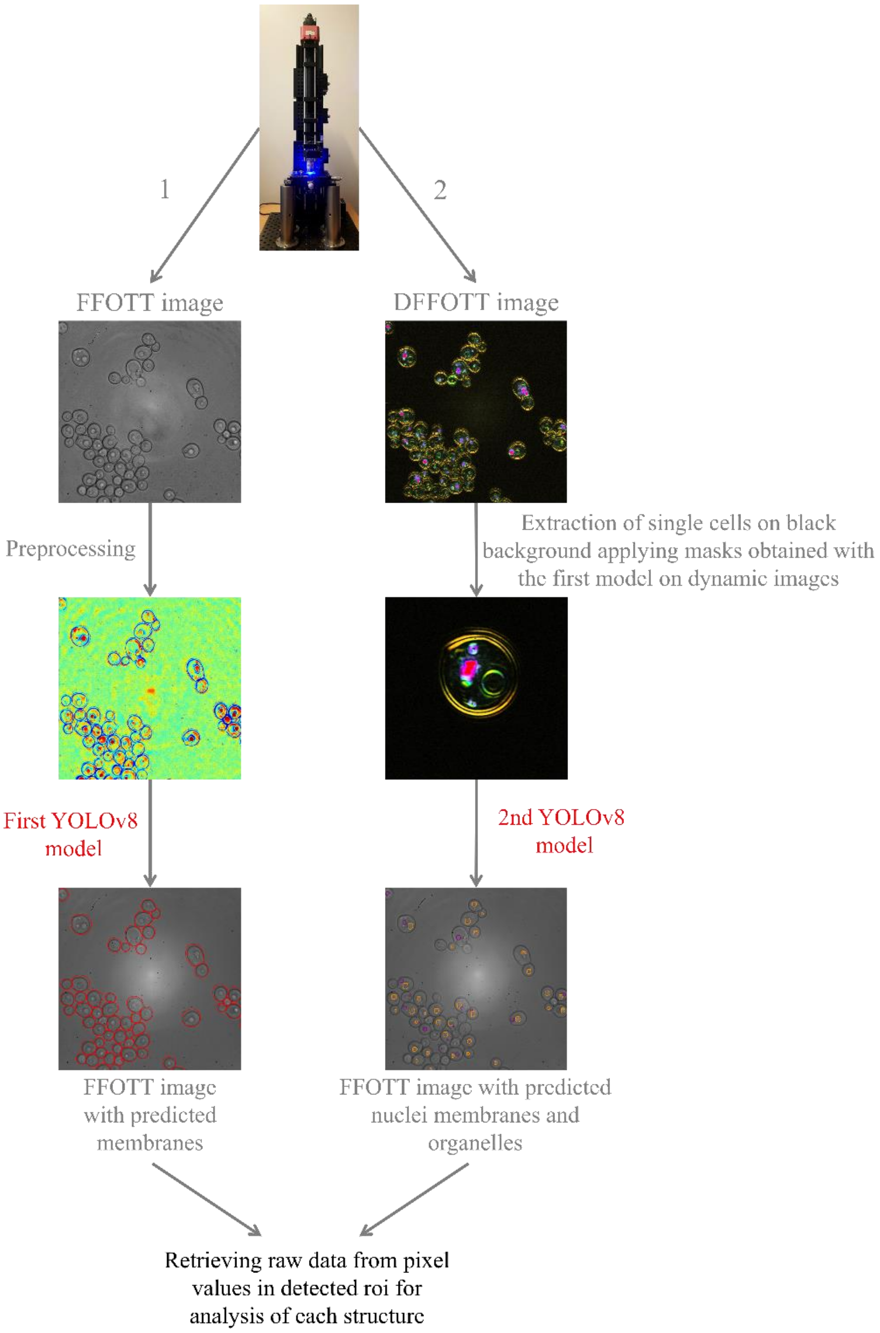

Figure S1: Schematic representation of a two-step neural network convolutional model for Candida albicans quantitative analysis.

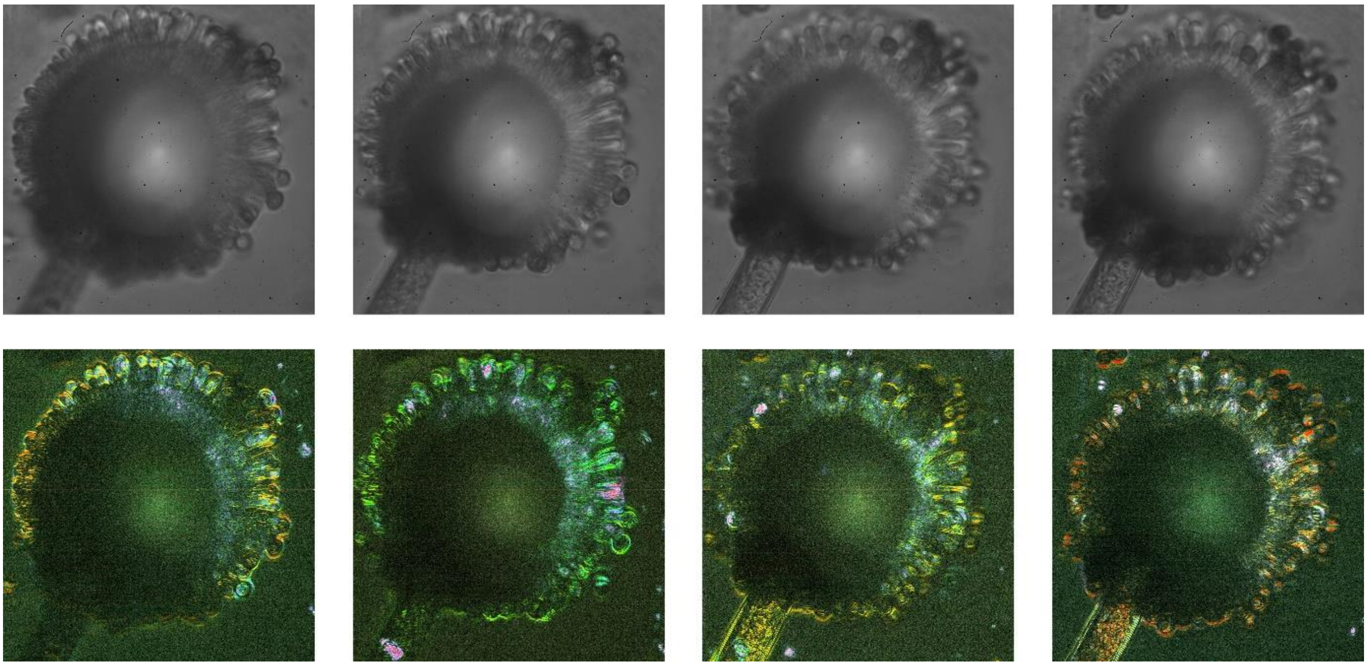

Figure S2: Typical images of an Aspergillus niger conidiophore realized at different depths showing no dynamic signal inside the vesicle and some bright pink signal inside few phialides or conidia.

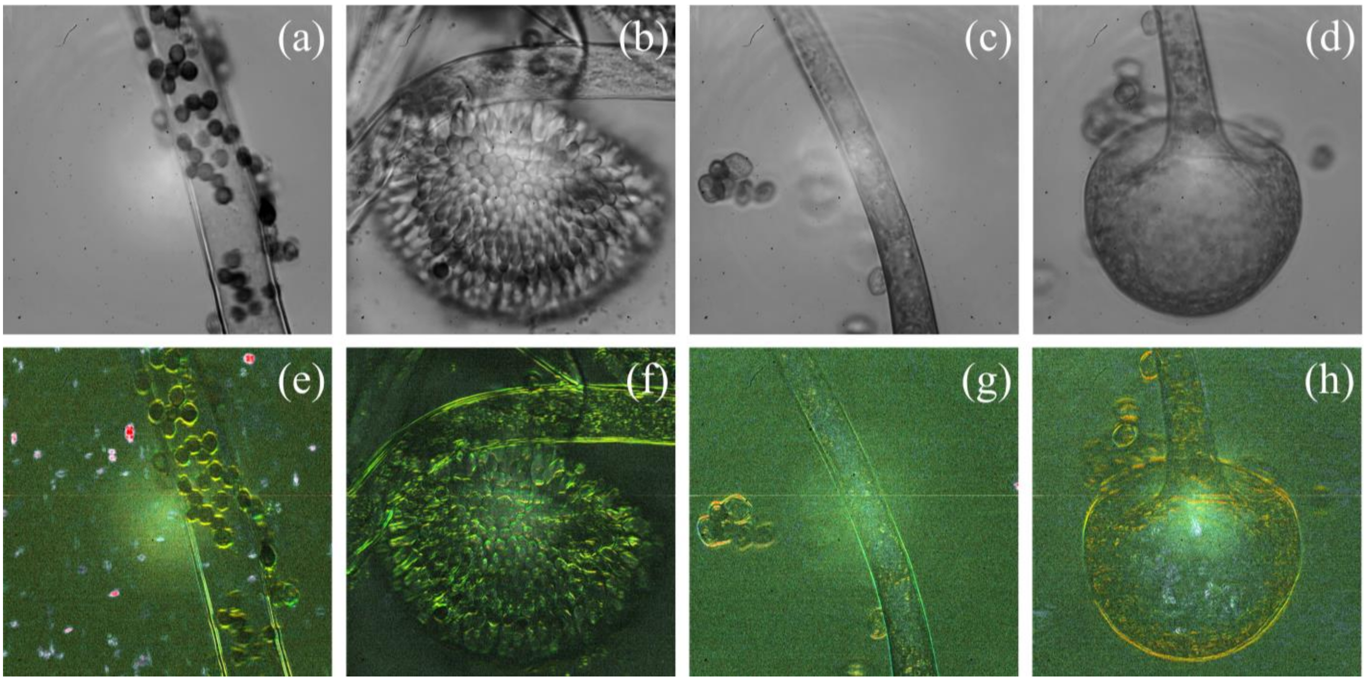

Figure S 3. D-FF-OTT of Aspergillus niger (a, b, e and f) and Rhizopus arrhizus (c, d, g and h) pretreated with lactophenol blue where hyphae, stipes and conidiophores have a green dynamic signal, close to the background noise.

Disclaimer/Publisher's Note: The statements, opinions and data contained in all publications are solely those of the individual author(s) and contributor(s) and not of MDPI and/or the editor(s). MDPI and/or the editor(s) disclaim responsibility for any injury to people or property resulting from any ideas, methods, instructions or products referred to in the content.
